# Neuropsychological insights into creativity in people with Parkinson’s disease

**DOI:** 10.1038/s41531-025-01165-y

**Published:** 2025-11-19

**Authors:** Sara Zeggio, David Steyrl, Matthew Pelowski, Paul Krack, Sirwan K. L. Darweesh, Julia S. Crone, Bastiaan R. Bloem, Marjan J. Meinders, Blanca T. M. Spee

**Affiliations:** 1 Department of Neurology, Radboud University Medical Center; Donders Institute for Brain, Cognition and Behavior; Center of Expertise for Parkinson & Movement Disorders, Nijmegen, The Netherlands; 2 Department of Cognition, Emotion, and Methods in Psychology, Faculty of Psychology, University of Vienna, Vienna, Austria; 3 Vienna Cognitive Science Hub, University of Vienna, Vienna, Austria; 4 Department of Neurology, University Hospital Bern, Inselspital, Bern, Switzerland; 5 Centre for Cognitive Neuroscience, Department of Psychology, Paris Lodron University Salzburg, Salzburg, Austria

**Keywords:** Human behaviour, Neurology, Neurological disorders, Neurodegenerative diseases, Parkinson's disease, Neurological manifestations

## Abstract

Creativity, the capacity and motivation to produce novel and personally meaningful ideas or behaviors, can be influenced by Parkinson’s disease (PD). Non-motor neuropsychological symptoms, such as apathy and negative schizotypy have been linked to reduced creativity, while dopaminergic treatments are associated with increased creative engagement. Building on epidemiological findings investigating changes in creativity, we examined possible drivers of increased and decreased creative activity. In a cross-sectional study, 360 participants with PD completed a questionnaire assessing self-reported creativity changes and associated factors, including personality (Big-Five, Multidimensional-Schizotypy-Scale), lifestyle (e.g., creative lifestyle, free time), and clinical (HY-scores, MoCA, dopaminergic treatments). Using machine learning (gradient-boosted decision-trees), we explained 23% of variance in creativity changes. Dopamine agonists, extraversion, free time, and a creative lifestyle since symptom onset predicted increased creativity, while disorganized schizotypy predicted decreases. The findings provide new insights for future research on creativity as part of PD’s neuropsychological spectrum and for person-centered treatment.

## Introduction

Parkinson’s disease (PD) is traditionally recognized for its motor symptoms^1^. However, PD also involves a range of cognitive and affective changes, including impaired executive functioning and mood disturbances, which significantly impact behavior in daily life^2–4^. Creativity—broadly defined as the motivation and behavior to engage in novel ideas or activities that offer both artistic expression and personal satisfaction^5^—has also been suggested to be influenced by PD-related symptoms as well as dopaminergic treatment^6–8^. This has been supported by a recent large-scale epidemiological survey, in which 41% of 793 individuals with PD reported to experience changes in their creativity, with a 2:1 ratio of decreases to increases^9^.

These changes may, in part, be explained by non-motor deficits, such as apathy, cognitive inflexibility and reduced novelty-seeking, which have been associated with decreases in both the capacity and motivation to engage in creative activities^10–12^. Studies suggest that a reduced engagement in artistic occupations prior to a PD diagnosis may reflect a decline in creative interest during the prodromal phase, with suggested potential recovery upon dopaminergic treatment^13–17^. Among dopaminergic treatment, dopamine agonists in particular have been associated with increased creativity^18–22^. Lifestyle adjustments following a PD diagnosis—such as shifts in artistic capacities and changes in creative style—have also been linked to increased inclination towards creative activities^15–17,23,24^.

These findings raise questions about whether changes in creativity, specifically the inclination to engage in and devote time to creative activities, are tied to the neuropsychological spectrum including changes in personality, lifestyle adjustments, and associated with clinical factors. Research on neuropsychological symptoms in PD highlights personality changes^20,25–27^. Such changes include, on the one end, reduced openness to experience (often associated with apathy), increased neuroticism and introversion, which are all linked to decreased creativity^28–30^. On the other end, traits like extraversion, novelty seeking, and positive schizotypy are associated with dopaminergic therapy and increased creativity^12,31,32^. Similar personality patterns have been observed in artists. Depressive states and negative schizotypy tend to correlate with reduced creativity, while manic states, extraversion, and positive schizotypy are linked to increased creativity^8,33–39^.

Lifestyle adjustments may also influence creativity from a neuropsychological perspective. A review on artistic changes in individuals with neurodegenerative disorders suggests that increased free time from early retirement may heighten the propensity to engage in creative activities^17,40^. Furthermore, the impact of a PD diagnosis on one’s personal life and social environment may encourage creative engagement, either as a personal coping strategy or through caregiver recommendations^18,41,42^.

We conducted an exploratory, data-driven investigation using machine learning to analyze the effects of personality traits, lifestyle adjustments, and clinical factors on creative engagement in individuals with PD. The analysis is based on data from 360 participants who took part in a follow-up to an epidemiological survey focusing on healthcare impact^43^ and creativity^9^. Our aim is to establish a foundation for hypothesis-driven research into the neuropsychological spectrum of PD, with a focus on creativity as a behavioral marker of disease impact, PD treatment, and lifestyle adjustments. By specifically highlighting creative behavior, our study may help inform person-centered care strategies.

## Results

Demographics and clinical characteristics are reported in Table 1. Our study involved a final sample of 360 participants (six participants were excluded from analysis due to incomplete questionnaire responses, i.e., not answering for any timepoint). Descriptive statistics and medication are reported in Table S1 in Supplementary Materials. Descriptive statistics of personality trait predictors as well as lifestyle adjustments and drug intake predictors are reported in Table S2 and S3 respectively. The majority of participants (89.25% on average over four items asking about arts training or education, Table S4 in Supplementary Materials) had no formal education in the arts.

**Table 1:** Demographics and clinical characteristics of the study population (*N* = 360)*

Demographics	
age—mean (SD) in years	69.0 ± 7.5
gender identity—*n* (%)	213 (59.2%) men
ethnicity—*n* (%)	357 (99.2%) Dutch
	3 (0.8%) other
educational level^a^—*n* (%)	72 (20.0%) low
	104 (28.9%) medium
	184 (51.1%) high
working situation^b^—*n* (%)	60 (16.7%) working*
	300 (83.3%) not working
living situation^c^—*n* (%)	53 (14.7%) living alone
	299 (83.1%) living with partner and children
	8 (2.2%) living in facilitated care
**Clinical characteristics**	
Hoehn & Yahr score^d^—*n* (%)^*^	
HY I: unilateral involvement	119 (33.1%)
HY II: bilateral involvement	122 (33.9%)
HY III: mild to moderate bilateral disability	62 (17.2%)
HY IV: severe disability	47 (13.1%)
HY V: wheelchair bound	4 (1.1%)
age: symptoms starting—mean (SD) in years	58.3 ± 11.6
age: diagnosis—mean (SD) in years	61.1 ± 10.6
global cognition^e^—mean (SD)	19.0 ± 2.5

^a^ Education was evaluated following Dutch school system standards into four categories, low education (in Dutch geen, basisonderwijs, VMBO, MAVO), medium education (in Dutch HAVO, VWO, MBO), higher education (in Dutch HBO, Universiteit, PhD), level unknown.

^b^ Working status was categorized as ‘working’ (full-time employment, part-time employment, self-employed, education following not paid by employer, voluntary work) or ‘not-working’ (retired, unemployed, disabled, sickness benefit, active in household).

^c^ Living situation used the three items: ‘alone’ (“I live alone.”), ‘with partner or family’ (“I live with my partner/my partner and children/family member other than partner”), or ‘in facilitated care’ (“I live in an institution/independently and receive outpatient supervision from a residential or welfare organization (assisted living)/a house belonging to a residential or welfare organization (sheltered living)).

^d^ Hoehn & Yahr (HY)-score followed the standard scale and parameters for physical severity of the disease, see for details Table S8 in Supplementary Materials.

^e^ Cognition was measures using the Montreal Cognitive Assessment (MoCA, short version, scale maximum 22 points).

*Missing data from six participants.

Descriptive statistics of reported engagement in creative activities, and changes therein, are reported in Table 2. Each creativity item showed responses across the full range of its respective scale. Participants reported a medium level of creativity before they remembered to notice symptoms (pre-symptomatic). We further observed that the proportion of participants self-reporting changes in creative activity—including both decreases and increases—grew over-time: from 52% at symptom-onset, to 61% post-diagnosis and 71% for current creativity. We further observed decreasing decrease-to-increase ratios (2.42:1 at symptom-onset, 1.91:1 post-diagnosis, 1.54:1 current creativity) suggesting a relative rise in increased creative activity over-time.

**Table 2:** Descriptive statistics of reported creativity change along the four time-points

Creativity	mean (SD)	no change n— (%)	decrease n— (%)	increase n— (%)
pre-symptomatic creativity *	6.6 ± 2.64			
symptom-onset creativity **	−0.4 ± 1.65	168 (47%)	133 (37%)	55 (15%)
post-diagnosis creativity ***	−0.4 ± 1.93	136 (38%)	143 (40%)	75 (21%)
current creativity	−0.3 ± 2.26	106 (29%)	154 (43%)	100 (28%)

*missing data from three participants.

** missing data from one participant.

***missing data from two participants.

Our study cohort showed relatively high levels for agreeableness and conscientiousness, while extraversion and openness were moderate to high^44^. Neuroticism was lower in comparison to Big Five psychometric norms^44^, though with more variability among participants. Our cohort exhibits higher levels of negative schizotypy (traits like social withdrawal and anhedonia), while positive schizotypy was less prominent based on the Multidimensional Schizotypy Scale (MSS)-B psychometric norms; disorganized schizotypy was just slightly higher than the reference sample^45–47^.

### Results of multivariate regression

The model’s performance was evaluated based on predicting current creativity changes at the time of the survey. The Mean Absolute Error (*MAE*) for the predictions was 1.48 ± 0.11, with a *p*-value of less than 0.001 when comparing the obtained *MAE* to that from shuffled labels, indicating a statistically significant result. The model’s Coefficient of Determination (*R²*) was 0.23 ± 0.10, with a corresponding *p*-value also less than 0.001 when compared to shuffled labels, further suggesting the model’s performance was significantly better than chance and indicating that the model explained 23% of the variance considering reported current creativity change.

**Fig. 1:**
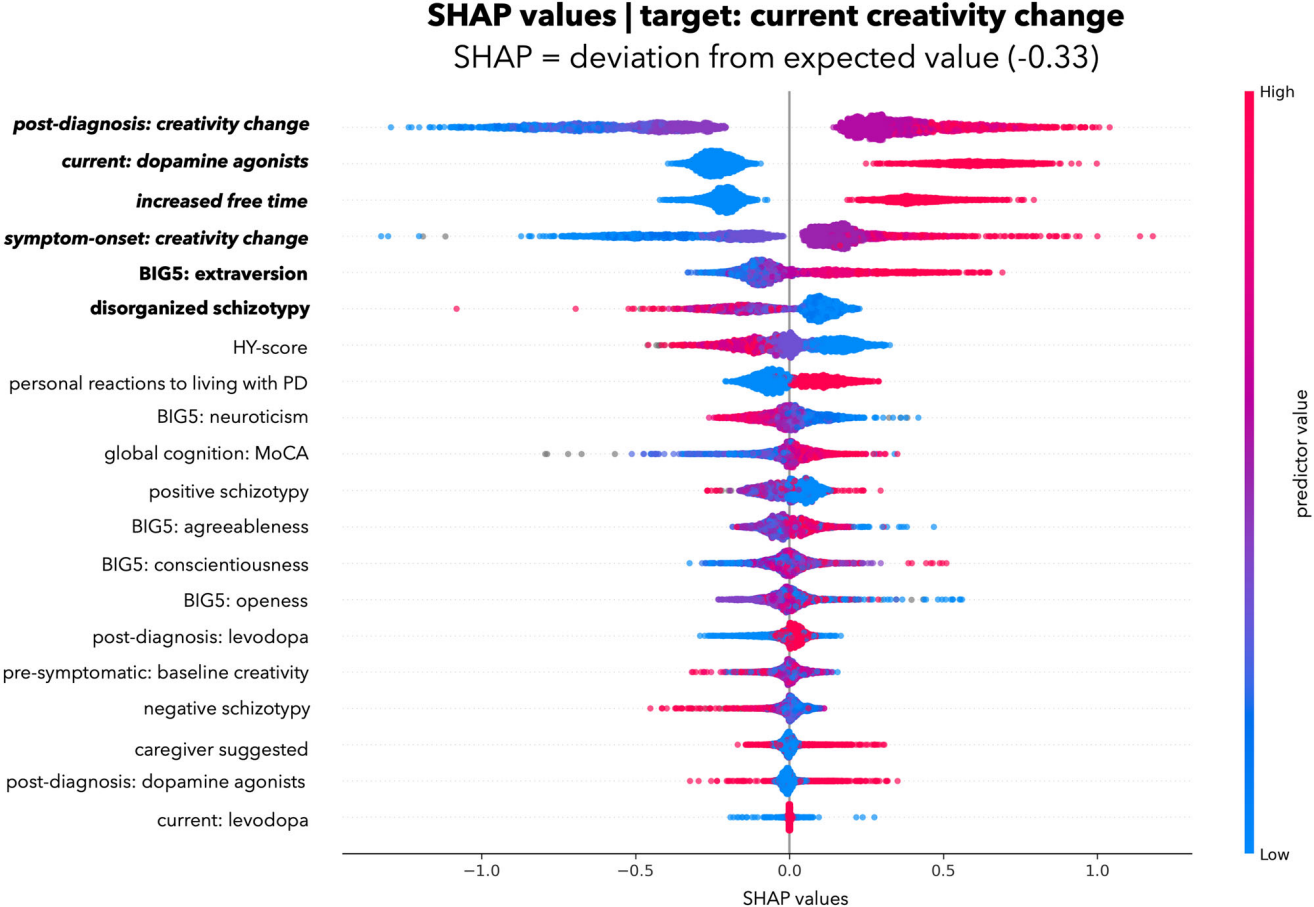
Individual factor importance for predicting current creativity change. Plot displays SHAP values (color code: low = blue, red = high, and showing non-linearity of associations). Note. This SHAP beeswarm plot visually summarizes the influence of predictors on a model’s predictions, illustrating both the magnitude and direction of their contributions. Each dot represents an individual data point, with the x-axis indicating the SHAP value (the predictors contribution to the prediction) and the color denoting the predictor’s value (e.g., blue for low values, red for high values). For the predictor post diagnosis: creativity change, low values (e.g., reduced post-diagnosis: creativity change) correspond to negative SHAP values, indicating a suppressive effect on the prediction of current creativity change. Conversely, high values (e.g., increased post-diagnosis: creativity change) are associated with positive SHAP values, signifying a promotive effect on the prediction.

**Fig. 2:**
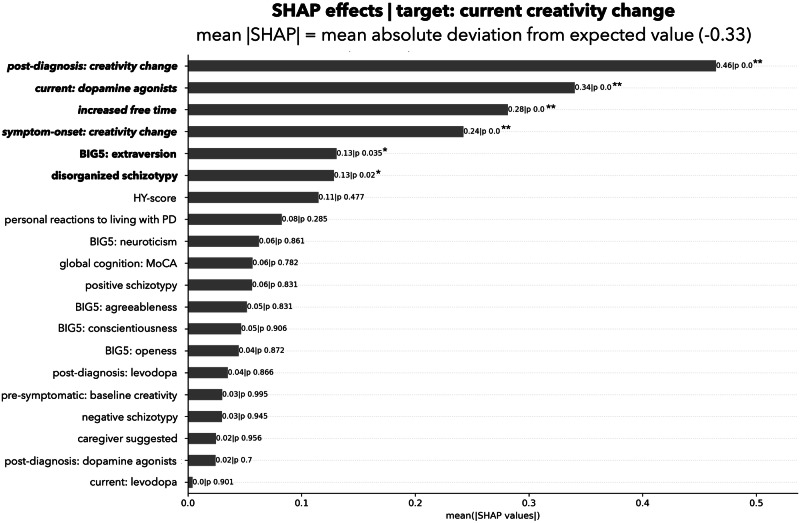
Rank based predictor importance based on mean absolute SHAP-value. Presented for each factor in association to the target: current creativity change; *p-value < 0.05 (factor highlighted by bold); **p-value < 0.05 incl. Bonferroni correction (factor highlighted by bold and italic).

We analyzed the contribution of the individual factors to the model’s performance, i.e., which factors are important to predict changes in creative activity. Figure 1 shows the individual factor importance for predicting creativity change. Figure 2 shows the rank-based predictor importance based on mean SHAP-values for each factor. Plots showing positive or negative association with the experienced creativity change of all significant factors are reported in Figs. S1–S6 in Supplementary Materials. Correlation heatmap is reported in Fig. S7 in Supplementary Materials). Full results, including how each predictor influenced the prediction results are available at our data depository: https://github.com/univiemops/creativity-parkinsons-disease.

The most important determinant of increased creativity came from the lifestyle adjustment category. Participants who were *creatively active after diagnosis* showed further increases in current creativity. The second significant factor, from the clinical factor category, was *current use of dopamine agonists*, which was positively associated with increased creativity. The third and fourth significant determinant, in the lifestyle adjustment category, was *increased free time* and *creativity at symptom onset* (where participant likely had not yet taken any medication for PD), again associated with increased creativity. In terms of personality traits, only two factors were significant: higher *extraversion* was associated with increased creativity, while higher *disorganized schizotypy* was linked to decreased creativity.

No other personality traits apart from extraversion and disorganized schizotypy contributed significantly to creativity changes. In the lifestyle adjustments category, *creatively active before symptoms* (pre-symptomatic) showed no significant effect. In the clinical factors, *current levodopa, earlier dopaminergic treatments (post-diagnosis)*, as well as *disease stage* and *cognition* showed no significant contributions.

We also analyzed SHAP interaction effects. Some interaction effects were found to be statistically significantly different from interaction effects obtained from models trained with shuffled prediction targets. However, the effect sizes were very small (~0.01) compared to statistically significant main effect sizes (lowest main effect about 10 times bigger than highest interaction effect). Furthermore, after multiple comparison correction no interaction effects remain significant. More details are available online: https://github.com/univiemops/creativity-parkinsons-disease.

## Discussion

Our analysis revealed significant contributions to reported current creativity changes in individuals with PD across all three of the selected categories––personality traits, lifestyle adjustments, and clinical factors. We found that engaging in a creative lifestyle after diagnosis, taking dopamine agonists, having increased free time, being creatively active upon noticing symptoms but before receiving a PD diagnosis, and the personality trait extraversion were connected to reported increased creativity. Higher disorganized schizotypy was associated with decreased reported creativity.

Considering our findings in personality traits and current creativity change, we found that extraversion was significantly associated with increased creative activity. The link of extraversion and creativity has been found in prior studies on professional and famous artists, where extreme levels also align with hypomania^12,48–50^. Extraversion’s link to creativity could also be partially attributed to its aspects of novelty seeking, risk-taking, and self-confidence—traits and states that have been connected to heightened dopaminergic activity and specific to hyperdopaminergic behavior within the behavioral spectrum of PD^10,19,20,22,51^. On the other hand, lower levels of extraversion have been reported in individuals with early PD and prodromal phases, such as those with rapid eye movement sleep behavior disorders^27^. This variation suggests that decreases and increases in active creative behavior, associated with extraversion levels, could serve as a more person-centered marker for identifying neurological changes linked to both the early stages of PD and response to dopaminergic treatment.

Contrary to established links wherein positive schizotypy correlates with increased creativity and negative schizotypy correlates with decreased creativity^31,52^, our findings showed that only disorganized schizotypy was associated with decreased creative activity. Even though our findings underscore the need for further research, we want to draw a potential connection between cognitive flexibility and divergent thinking skills necessary for productive creative activity^35,53,54^. Prior literature suggests that creative cognition is dependent on moderate (but not low or high) levels of striatal and pre-frontal dopamine, which has also been linked to cognitive flexibility, divergent thinking, and problem-solving capabilities^21,55^. These cognitive attributes appear to be crucial for successful creative behavior (ideation/imagination, production, revision/refinement, and personally useful/socially acceptable products/activities^39,55–57^). In individuals with PD, they have been observed to fluctuate, possibly linked to variations in dopaminergic activity^10,21,58,59^. The association between disorganized schizotypy and reduced creativity could therefore reflect disruptions in these cognitive processes and neurological balance, which are vital for both generating and executing creative ideas^35,54^. We acknowledge that more studies remain necessary to explore how disorganized schizotypy affects creative behavior in individuals with PD and its potential links to dopaminergic modulation, before making any further implications.

Our findings in lifestyle adjustments revealed a significant positive association between increased free time and increased creative activity. This finding emphasizes the influence of leisure activities on creativity among individuals with PD, also suggesting that investing in a creative lifestyle might mitigate the disease’s impact on daily life and, potentially increase naturally dopaminergic activity^20,21^ and consequently well-being^18^. Regardless, allocating more free time to creative activities post-diagnosis, might well be regarded as another “silver lining” of having received this serious and life-altering diagnosis^40^.

Interestingly, a pre-symptomatic creative lifestyle did not predict either increased or decreased activity, suggesting that early creative habits alone may not influence how creativity evolve throughout life and later during disease course. However, our results did reveal that creativity tended to change around the time of symptom-onset and increased further after diagnosis, with decreasing decrease-to-increase ratios over-time. These findings may complement recent epidemiological studies that have reported a lower prevalence of PD among individuals in artistic professions later in life^13,14^. Rather than implying a protective effect of artistic work, these patterns may reflect differences in dopaminergic function prior to diagnosis. For example, sustained artistic engagement in later life may indicate a still-functioning mesolimbic dopaminergic system. This is consistent with findings in nicotine research, where individuals with PD often stop smoking years before motor symptoms due to reduced dopaminergic reward sensitivity^60^. Similarly, early dopaminergic decline may affect personality traits, such as novelty seeking of artistic drive, influencing career choices long before diagnosis.

Nevertheless, engaging in creative activities after diagnosis may still serve as a valuable coping strategy. Such activities can stimulate neurotransmission and help preserve or strengthen synaptic function in meso-cortical circuits, particularly important in individuals with PD, where dopamine-related cognitive and affective circuits are affected. Tailored programs aligned with personal interests and abilities^61,62^ may support individuals in maintaining or re-engaging with creative pursuits when other occupational or recreational avenues are no longer feasible^24,42^.

Among the clinical factors, our findings underscore and align with previous research on the significant influence of current medication intake, rather than long-term or past usage, in changing neuropsychological behavior in PD^4,11,12,19,20^. In our study, this was particularly evident with dopamine agonists (which have a higher affinity to the mesolimbic dopamine D3 receptors compared to endogenous dopamine synthetized from levodopa) in relation to increased creative activity. These results suggest that adjustments to dopaminergic treatment could have a rather immediate effect on creative behavior, offering a potential person-centered indicator of drug efficacy that might guide dosage modifications to optimize therapeutic outcomes. However, our findings offer significant implications only when considering parallel neuropsychological assessments in PD and within-subject comparisons of experienced creativity change. Currently, few neuropsychological scales consider changes in creative behavior as a marker for medication-induced alterations in behavior. Notably, the Ardouin Scale, which assesses both hyper- and hypodopaminergic behaviors in PD, is one of the few tools that includes creativity as an evaluative component^51,63^. Within this scale creativity is suggested to be part of impulse control and related disorders that share common underlying mechanisms but needs to be separated from hobbyism and punding using comprehensive clinical criteria^51,63,64^. Our findings, alongside assessment tools, such as the Ardouin Scale, highlight the need for broader integration of creative behavior and changes thereof into clinical assessments as an important aspect of understanding medication effects in PD management.

Increased creativity related to dopaminergic treatment can result not only from disinhibition of previously existing cognitive abilities (such as high intelligence quotient, working memory skills, cognitive flexibility, divergent thinking) and mood (change towards hypomania), but also from modification in personality traits (such as increased novelty seeking, positive schizotypy) in individuals with PD. Creative activity can only occur in a prepared mind, and dopaminergic neurotransmission, although capable of facilitating creative drive, cannot create creative intelligence but rather impact the three basal ganglia-thalamo-cortical loops by enhancing cortico-cortical associations, bottom-up appetitive drive and decreasing prefrontal top-down behavioral control^19,31,50,65–67^.

The distinct impact of dopamine agonists, predominantly D2/D3 receptor selective (notably, nine out of 101 participants took apomorphine that impacts both D1 direct and D2 indirect dopaminergic pathways, hence acting similar to levodopa), contrasts sharply with the lack of prediction importance of levodopa. This discrepancy raises key questions about the specific neurochemical pathways that modulate creative behavior within the dopaminergic system^10,21,68^. The currently available dopamine agonists—with the notable exception of apomorphine—have a high affinity to the mesolimbic D3 dopamine receptor, which helps explain their dose-dependent potential to induce impulse control disorder and related disorders^65,69^. In contrast, the effects of apomorphine and levodopa on these behaviors are more closely tied to sensitization of the dopaminergic system. This sensitization is not merely dose-dependent but also significantly influenced by the severity of the presynaptic dopaminergic neurodegeneration underlying pulsatility of the treatment with levodopa^60,70^. Apomorphine further contributes to pulsatility due to its pharmacokinetic properties—being lipophilic with rapid resorption after injection into subcutaneous fat, easily crossing the blood brain barrier, and having a short half-life. This pharmacodynamic profile may explain why apomorphine more frequently leads to dopamine dysregulation syndrome, which is thought to result from rapidly induced psychotropic effects mediated in the dopaminergic synapse at the level of the nucleus accumbens^71^.

In our study, levodopa was not significantly associated with reported changes in creativity. This is likely related to the fact that our sample predominantly included individuals with moderate disease stage and not with advanced PD. Indeed, in populations representative of the general PD population, dopamine agonists are a main risk factor to develop impulse control disorder and related behaviors^72^. In contrast, in advanced PD populations—particularly those undergoing advanced treatments, such as deep brain stimulation—motor complications and impulse control disorder have been observed even in absence of dopamine agonists^4,12^. This distinction highlights the importance of disease stage, treatment context, and pharmacological specificity in interpreting the behavioral effects of different dopaminergic therapies.

Our findings raise important ethical considerations in clinical practice. While dopamine agonists may facilitate beneficial changes, such as increased creative engagement, they also carry risks of maladaptive behaviors, including impulse control disorders^4,73^. Clinicians should therefore evaluate both the therapeutic potential and the neuropsychological side effects of dopaminergic medications, ideally within a framework of shared decision-making, regular behavioral monitoring and expert guided creative engagement when given as a treatment and coping strategy^40,62^.

A limitation of our study is the retrospective self-reported nature of experiencing changes in creative activities, which may be subject to recall bias and potential anchor effects, particularly given the use of similarly scaled items across different time points. While the outcome and key predictor refer to distinct phases—current engagement and post-diagnosis adjustment—their conceptual proximity may have influenced participants’ responses and affect the reliability of our data. Disease severity and cognition were evaluated using questionnaires assessed through an assessor during the initial baseline PRIME survey^43^. Gender identity was not included as a predictor in the current model, although exploratory findings in another study from the larger PRIME cohort suggest it may be relevant for future analyses^9^. Additionally, the use of machine learning models on a rather small sample size may limit the generalizability of our findings. It is crucial to interpret our results within the context of the moderate amount of explained variance and we should remain cautious about extrapolating these findings to the broader PD population without further study.

Despite these limitations, our exploratory and data-driven approach provides a valuable new perspective on neuropsychological phenomena associated with PD. Future research should aim to validate these findings in larger, prospective studies looking at individual changes in creative activities and creative cognition skills from prodromal to early onset and later drug efficacy. Further hypothesis-driven studies could provide new insights to our findings, especially those on personality traits, dopaminergic treatment effects, lifestyle, and creative activity as part of changed neuropsychological behavior. This could further inform the development of diagnostic tools and patient care strategies that incorporate the neuropsychological and creative engagement characteristics of individuals with PD, ultimately contributing to the advancement of person-centered medicine.

## Methods

We conducted an exploratory cross-sectional study using self-reported questionnaires to collect data once per participant from March 2021 to March 2022.

### Participants

Inclusion criteria comprised a clinical diagnosis of parkinsonism, excluding drug-induced parkinsonism, in patients who had visited a Neurology outpatient clinic at one of the four community hospitals associated with the previously described ‘Proactive and Integrated Management and Empowerment in PD (PRIME Parkinson) healthcare innovation project in the South-East region of the Netherlands^43,74^. In short, the PRIME Parkinson project consists of the implementation and evaluation of a new care model for people with PD entitled ‘PRIME Parkinson’ care.

### Procedure

We invited participants who are enrolled in the PRIME-NL study, which evaluates the new PRIME Parkinson care model^43^. The PRIME-NL study is coordinated through the Radboudumc (Center of Expertise for Parkinson and Movement Disorders).

Our initial survey study included 793 respondents from 913 participants within the PRIME-NL cohort^9,43^. Out of these, we invited participants to complete an additional one-time questionnaire on experienced changes in their creativity and associated factors as described in study endpoints between March 2021 to March 2022. Of those who attended the current study, 45.9% reported experiencing a change (increase/decrease), while 54.1% had reported not to have experienced a change in creativity in the initial survey^9^. Comparisons of invitees and attendees along demographic, lifestyle factors, and healthcare region provided by PRIME Parkinson are provided in Table S5-S7 in Supplementary Materials. Eligible participants received an information letter outlining the study’s background, objectives, and methods. Following this, they had the opportunity to discuss any questions with the researcher to fully understand the implications of their involvement. Those who agreed to participate provided written informed consent. Participants received, based on individual preference, either a digital survey version (SoSci-Survey^75^) or a paper-pencil version; people were asked to complete the survey within two weeks with two weeks extension in case of request.

Our study was approved by the ethical board METC Oost-Nederland, Radboudumc (CMO file numbers 2021-12985). Participants had to be willing and able to provide written informed consent.

### Study endpoints

We developed a predictive model for our dependent variable, termed ‘current creativity change,’’ which reflected self-experienced changes in engaging into creative activities. Participants were asked to consider their momentary (at timepoint of survey) experience, including their creative engagement over the past three months. The possible range of creative activities was explained by providing examples across diverse creative domains, including visual arts, performing arts, literature, and music^76^. Additionally, we included complementary arts, such as creative cooking, science and technology, handicrafts, interior and garden design, and other practical crafts^77^ (see for full detail of wording description D1 in Supplementary Materials). This variable was assessed using an 11-point Likert scale, with responses ranging from -5 (strong decrease) to +5 (strong increase), with 0 indicated no change.

We aimed to explore a comprehensive list of potential factors; however, constraints of the data-driven model and the number of eligible participants limited us to including a maximum of twenty independent variables. Therefore, we focused on three key factor categories:

Personality-related factors were represented by eight determinants using two instruments measuring traits. This included the 10-item version of the BIG5 dimensions^44^ (5-point Likert scale; strongly disagree to strongly agree): (1) openness to experience, (2) conscientiousness, (3) extraversion, (4) agreeableness and (5) neuroticism. We used the Multidimensional Schizotypy Scale (MSS-B)^45–47^ with a binary scale (true, not true) to determine schizotypy along three sub-scales: (6) *positive schizotypy* (or psychotic-like symptom) dimension is characterized by disruptions in the content of thought, ranging from magical ideation to full-blown delusions, perceptual oddities (including illusions and hallucinations), and suspiciousness/paranoia; (7) *negative schizotypy* (or deficit) dimension involves a diminution in experiences, encompassing symptoms, such as alogia, anergia, avolition, anhedonia, flattened affect, and disinterest in others and the world; (8) *disorganized schizotypy* (or cognitive-behavioral disorganization) dimension is characterized by disturbances in the ability to organize and express thoughts and behavior, ranging from mild disruptions in thinking and behavior to formal thought disorder and markedly disorganized actions. Due to the absence of a validated Dutch version, the MSS-B items were translated and back-translated from the original version following the cross-cultural adaptation guidelines^78,79^.

Lifestyle adjustments was represented by six determinants. *Creative lifestyle* was assessed for three time points (self-reported, retrospectively, see also Description D1 in Supplementary Materials): (1) *pre-symptomatic baseline* (assessed whether the participant was creatively active before noticing any symptoms, 11-point Likert scale from 0 = ‘not at all creatively active’’ to 10 = ‘very much creatively active’’), (2) *creativity change at symptom onset* (examined whether the participant was creatively active upon noticing symptoms but before receiving a PD diagnosis), and (3) *creativity change post-diagnosis* (evaluated whether the participant to engaged in creative activities after receiving a diagnosis). The last two factors were evaluated on a 11-point Likert scale ranging from -5 (strong decrease) to +5 (strong increase), with 0 indicated no change. Participants were also asked why they believe their creativity had or had not changed, using a binary scale (yes/no). Included factors were that participants experienced a change due to (4) *increased free time*, (5) *personal reactions to living with PD*, and (6) *caregiver suggested*; latter meant that either family/friends or healthcare professional had recommended the person with PD to engage in creative activities.

Clinical factors included six determinants: (1) the *disease stage* as defined by the Hoehn-Yahr (HY) score and based on six questions from the Movement Disorder Society Unified PD Rating Scale (MDS-UPDRS^80^) and two self-structured items (see Table S8 for HY-score categorization parameters); (2) *cognition* was assessed using the short version of the Montreal Cognitive Assessment (MoCA) (MoCA^43,81^, scale maximum 22 points). Data on disease stage and cognition were assessed by the initial PRIME baseline survey through an assessor^43^. Finally, *dopaminergic treatment* was assessed focusing on two self-reported timepoints: (3) *post-diagnosis medication* (immediately after diagnosis) and (4) *current medication* (over the past three months at the timepoint of answering the questionnaire). We split the medications further into two categories, (5) *levodopa only*, and (6) *dopamine agonists*, *with or without levodopa intake* (see for drug lists, medication classes, and descriptive statistics Table S1 in Supplementary Materials).

### Data analysis: multivariate regression

We adopted an interpretable machine learning-based approach for multivariable statistical analysis^82,83^. The multivariable statistical regression analyses were conducted using Python v3.11 and the scikit-learn library v1.4^84^. The process involved three main steps: (1) training prediction models, (2) testing generalizability, and (3) analyzing the models.

Prediction Model Training: Gradient Boosted Decision Tree (GBTD) models were chosen for the regression task due to their computational efficiency and high accuracy^85,86^. GBDT models inherently capture non-linear associations and variable interactions. They are robust against multicollinearity and outliers in the data. The 20 predictors for these models included clinical factors, personality-related factors, and lifestyle adjustments with ‘current creativity change’ serving as the prediction target.

Generalizability Testing: To evaluate the model’s ability to generalize to unknown (out-of-sample) data, a nested cross-validation (CV) procedure was implemented, as recommended by prior research^87,88^. This procedure involves repeated splits of the data into training and testing sets. In the outer CV loop a 5-folds split scheme grouped by participants was applied repeatedly until a minimum number of 10,000 predictions was reached. Model complexity tuning utilizes a random search scheme to identify well performing complexity parameters (column sample per tree 0.1–1; using extra trees True/False; path smoothing 1–100 log-scale). The performance of each parameter set was assessed in a nested (inner) CV procedure, using training data only. A 5-folds split scheme grouped by participants was applied again and repeated until a minimum number of 1000 predictions was met. The parameter set achieving the highest score was selected and subsequently employed in the model of the main CV loop along with constant parameters (learning rate 0.01; number of leaves 100; number of boosting rounds 1000). Regression performance was measured using prediction coefficient of determination score (prediction *R²*) and MAE score, while classification performance was evaluated using balanced classification accuracy score^89^.

Model Analysis: The importance of individual predictors, i.e., the effect on the prediction, was determined using SHapley Additive exPlainations (SHAP), an interpretable machine learning method based on cooperative game theory^90,91^. SHAP assesses each predictor’s contribution to the model’s performance, providing a detailed analysis of their significance in the regression task.

### Data analysis: statistical tests

The assessment of statistical significance for differences between the means of prediction *R²* metric, prediction accuracy, and the importance of predictors was carried out using a modified paired *t*-test. It compares the observed values with their counterparts generated under a simulated null-hypothesis, where data labels are subjected to shuffling. To address sample dependence introduced by CV, the *t*-test was adapted, aligning with the recommendations of prior research^92,93^. A Bonferroni correction was applied to the *t*-test results in instances where multiple comparisons were conducted. We provide full access to the data and results: https://github.com/univiemops/creativity-parkinsons-disease.

### Sample size considerations

Our study was exploratory, and without a preceding study to guide a power analysis, we opted for a convenience sample. Drawing on prior experience with PRIME-NL participants, we estimated that at least 250 individuals from the available recruitment cohort would participate (comparisons of invitees and attendees along demographic, lifestyle factors, and healthcare region provided by PRIME Parkinson are provided in Table S5–S7 in Supplementary Materials).

Determining an adequate sample size for non-linear machine learning models is challenging, as no established standards exist for high-dimensional, multivariable analyses^94^. General guidelines suggest requiring 10–20 samples per predictor or a minimum of 50 samples to initiate meaningful machine learning analysis^84^. Another contentious viewpoint advocates for a minimum of 10–20 samples per degree of freedom (predictor), potentially resulting in a range of 200–400 samples for the current study^95,96^. Our final dataset included 360 participants, after excluding six cases due to recording issues. This sample size reflects a pragmatic balance between methodological recommendations and resource constraints. Based on a post hoc power analysis, the study achieved a statistical power of 76.4% to detect small effects (Cohen’s *d* = 0.2) at a significance level of 0.05, which approaches the conventional threshold of 80%.

## Supplementary information

Supporting Information

## Data Availability

The datasets generated and/or analyzed during the current study are available in the github repository: https://github.com/univiemops/creativity-parkinsons-disease.
