# Supplementary material for: Neuropsychological insights into creativity in people with Parkinson’s disease
Source: NPJ Parkinsons Dis. 2025 Nov 19;11:324. doi: 10.1038/s41531-025-01165-y (PMC12630753; doi:10.1038/s41531-025-01165-y)
Supplement: Supplementary file 1 — Supporting Information [file 41531_2025_1165_MOESM1_ESM.pdf]

Supplementary Material

Neuropsychological Insights into Creativity in People with Parkinson's Disease

Sara Zeggio[1], David Steyrl[2], Matthew Pelowski[2,3], Paul Krack[4], Sirwan K.L. Darweesh[1], Julia S. Crone[3], Bastiaan R. Bloem[1], Marjan J. Meinders[1], Blanca T.M. Spee[1,2,3,*]

[1] Department of Neurology, Radboud University Medical Center; Donders Institute for Brain, Cognition and Behavior, Center of Expertise for Parkinson & Movement Disorders, Nijmegen, The Netherlands

[2] Department of Cognition, Emotion, and Methods in Psychology, Faculty of Psychology, University of Vienna, Vienna, Austria

[3] Vienna Cognitive Science Hub, University of Vienna, Vienna, Austria

[4] Department of Neurology, University Hospital Bern, Inselspital, Bern, Switzerland

* Corresponding Author: blanca.spee@univie.ac.at, blanca.spee@radboudumc.nl

Descriptions

Description D1.

Participants were asked with the following description:

“We will ask questions about both your feelings of being creative and your creative activities. We also want to know how your feeling of being creative and your creative activities might have changed.

We will ask 4 time points:

I. BEFORE you noticed any Parkinson's disease related SYMPTOMS.

II. AFTER you noticed Parkinson's disease related SYMPTOMS, but BEFORE your DIAGNOSIS.

III. AFTER your DIAGNOSIS with Parkinson's disease.

IV. In the LAST 3 MONTHS until NOW.”

For each timepoint we asked individually on an 11-Point Likert scale:

I. ***“Before** I noticed Parkinson’s-related **symptoms** in myself, I was:” “never doing anything creative”* to *“very often doing something creative;”* Likert Scale from 0-11

II. ***“After** I noticed the first Parkinson’s-related **symptoms** in myself, I noticed that __**my creative activities**__:”* “*decreased*” (-5 to -1) to “*increased*” (+1 to +5); 0 represented stability (no change)

III. “**After** my **diagnosis** with Parkinson’s disease, I noticed that __**my creative activities**__**:**” “*decreased*” (-5 to -1) to “*increased*” (+1 to +5); 0 represented stability (no change)

IV. ***“In the last 3 months until now**, I have noticed that __**my creative activities**__:*” “*decreased*” (-5 to -1) to “*increased*” (+1 to +5); 0 represented stability (no change)

Participants were given several ideas in which creative domain they could have been active:

- Visual art (e.g., drawing, creative photography, sculpturing, etc.)
- Performing art (e.g., playing theatre, dance, etc.)
- Literature (e.g., writing texts, blogs, poems, etc.)
- Music (e.g., composing or adapting melodies)
- Handicraft (e.g., making own cards, cloths, bags, etc.)
- Interior and garden design (e.g., designing/embellishing one’s living space)
- Creative cooking (e.g., creative novel dishes/drinks)
- Science/Technology (e.g., solving technical problems, computer programming, etc.)
- Social (e.g., inventing games, organizing parties, etc.)

Tables

Table S1. Categories of drugs and medication classes, including absolute numbers of reported intake.

Index	Brand	Category	*n* (post-diagnosis)	*n (*current*)
1	Levodopa/carbidopa – Sinemet	levodopa	157	305
2	Levodopa/benserazide – Madopar	levodopa	84	180
3	Levodopa/carbidopa – Duodopa	levodopa	115	130
		levodopa total	226	337
4	Pramipexol – Mirapex, Glepark, Sifrol	dopamine-agonist	23	90
5	Ropinirol – Requip	dopamine-agonist	23	45
6	Rotigotine – Neupro = transdermal patch	dopamine-agonist	3	42
7	Apomorphine (APO-go, Dacepton)	dopamine-agonist	1	9
8	Bromocriptine (Parlodel)	dopamine-agonist	0	1
9	Pergolide	dopamine-agonist	1	0
		dopamine agonist total	50	101

Note: Due to lack of supporting literature, we did not separate between enteral levodopa and levodopa administered intermittently; participants could take multiple drugs within same category. All participants took either levodopa only (categories indices 1-3) or dopamine agonists (category indices 4-9); in latter group, most participants also took levodopa (only four reported taking dopamine agonists only); *current means at timepoint of survey including the last three months.

Table S2. Descriptive statistics of personality traits predictors (*N* = 360).

**Personality traits**	**Mean (*SD*)**
BIG5_extraversion *	6.48 (±1.61)
BIG5_agreeableness **	7.29 (±1.17)
BIG5_conscientiousness*	7.46 (±1.53)
BIG5_neuroticism **	5.77 (±1.83)
BIG5_openness **	6.43 (±1.73)
positive_schizotypy ***	0.99 (±1.42)
negative_schizotypy ***	2.49 (±2.26)
disorganized_schizotypy ***	1.90 (±2.65)

Note. *missing data from 4 participants; **missing data from 3 participants; ***missing data from 8 participants.

Table S3. Descriptive statistics of lifestyle adjustments and drug intake predictors (N = 360).

Factors	No, *n*—(%)	Yes, *n*—(%)
personal reaction to PD	215 (60%)	145 (40%)
caregiver suggested *	269 (74%)	90 (25%)
increased free time	238 (66%)	122 (34%)
post-diagnosis levodopa	94 (26)	266 (74%)
post-diagnosis dopamine agonist	310 (86%)	50 (14%)
current levodopa	23 (6%)	337 (94%)
current dopamine agonist	259 (72%)	101 (28%)

Note. *missing data from one participant.

Table S4. Level of art education in study cohort (*n* = 349).

Arts training or education	none	a few (max 3 courses)	some courses as hobby	some courses as training/education	graduation in study
theoretical education in fine arts	319 (91.4%)	4 (1.1%)	12 (3.4%)	4 (1.1%)	10 (2.9%)
education in art history	315 (90.3%)	7 (2.0%)	10 (2.9%)	12 (3.4%)	5 (1.4%)
practical education in arts	295 (84.5%)	9 (2.6%)	24 (6.9%)	9 (2.6%)	12 (3.4%)
other education in arts	317 (90.8%)	2 (0.6%)	18 (5.2%)	4 (1.1%)	8 (2.3%)

Note. 11 participants filled in less than two out of four responses and were excluded from the analysis

Table S5. Demographic and lifestyle descriptive statistics of participants attending our study from the PRIME Parkinson cohort (*N* = 913 eligible participants invited to our study).

	**attended**		**did not attend**		**95% CI**			
Factor	***N* (%)**	**mean ± *SD***	***N* (%)**	**mean ± SD**	**Lower**	**Upper**	***p*-value**	***t*-test**
age	365 (39.98%)	69.05 ± 7.50	548 (60.02%)	71.07 ± 8.16	-3.07	-0.97	< .001	-3.79
cognition[a,*]	361 (40.20.%)	18.69 ± 2.68	537 (60.80%)	17.71 ± 3.01	0.61	1.36	< .001	5.13
HY-score[b,*]	360 (40.04%)	2.16 ± 1,07	539 (59.96%)	2.55 ± 1.24	-0.54	-0.23	< .001	-4.98
disease duration	365 (39.98%)	5.90 ± 4.14	548 (60.02%)	6.30 ± 5.28	-1.01	0.22	.205	-1.27
creatively active prior PD[c,*]	364 (40.18%)	4.31 ± 1.96	542 (59.82%)	3.56 ± 2.03	0.49	1.02	< .001	5.58

Note. Results show that younger participants, those with higher cognitive function (MoCA), lower HY-scores, and who were more creatively active before PD were more likely to attend in our study. *Level of being creatively active before PD was measured in the PRIME Parkinson survey using a 7-Point Likert scale from 1 = not at all, 7 = very much). [a.] Cognition was measures using the Montreal Cognitive Assessment (MoCA); [b.] Hoehn & Yahr (HY)-score followed the standard scale and parameters for physical severity of the disease, see for details Table S1; [c] creative lifestyle score are based on 7-point Likert-type scale (“*Before my diagnosis with PD, I participated in creative activities?”* 1 = “not at all,” 7 = “very much”).*Note, missing data from several participants.

Table S6. Demographic and lifestyle descriptive statistics of participants attending our study from the PRIME Parkinson cohort, (*N* = 913 eligible participants invited to our study).

factor	attend<br>N (%)	did not attend<br>N (%)	df	χ^2 Value	*p*-value
gender	217 (59.5%) men<br>148 (40.5%) women	335 (61.1%) men<br>213 (38.9%) women	1	0.26	.611
ethnicity	363 (99.5%) Dutch<br>2 (0.5%) other	542 (98.9%) Dutch<br>6 (1.1%) other	1	0.76	.385
education[a,*]	74 (20.3%) low education<br>103 (28.2%) medium education<br>186 (51.0%) high education	152 (27.7%) low education<br>138 (25.2%) medium education<br>256 (46.7%) high education	3	6.68	.083
working status[b]	305 (83.6%) not working<br>60 (16.4%) working	459 (83.8%) not working<br>89 (16.2%) working	1	0.01	.937
living situation[c]	52 (14.2%) alone<br>304 (83.3%) with partner/family<br>9 (2.5%) facility	73 (13.3%) alone<br>464 (84.7%) with partner/family<br>11 (2.0%) facility	2	0.40	.820
creativity change[d,*]	167 (45.9%) yes<br>197 (54.1%) no	161 (29.7%) yes<br>381 (70.3%) no	1	24.67	<.001

Note. Results show that people who reported a change in creativity in the PRIME Parkinson initial survey were more likely to attend our study. [a.] Education was evaluated following Dutch school system standards into four categories, low education (in Dutch geen, basisonderwijs, VMBO, MAVO), medium education (in Dutch HAVO, VWO, MBO), higher education (in Dutch HBO, Universiteit, PhD), level unknown. [b.] Working status was categorized as ‘working’ (full-time employment, part-time employment, self-employed, education following not paid by employer, voluntary work) or ‘not-working’ (retired, unemployed, disabled, sickness benefit, active in household). [c.] Living situation used the three items: ‘alone’ (“I live alone.”), ‘with partner or family’ (“I live with my partner/my partner and children/family member other than partner”), or ‘in facilitated care’ (“I live in an institution/independently and receive outpatient supervision from a residential or welfare organisation (assisted living)/a house belonging to a residential or welfare organisation (sheltered living). [d]To report overall creativity change, participants responded to: *“Have you noticed changes in your creativity or desire to engage in creative activities related to life with Parkinson’s disease? This may include feeling more or less creative or feeling the need to be creative (e.g., painting, drawing, writing, music, dancing, photography, gardening, sewing, etc.)”* (translated from Dutch by the authors). *Note, missing data from several participants.

Table S7. Descriptive statistics of creativity change along healthcare regions* (*N* = 360).

**Region of Healthcare**	
PRIME region—no. (%)	97 (26.94)
Usual care region—no. (%)	263 (73.06)
**Reported current creativity change along healthcare region***	
Reported decrease in PRIME-NL region—no. (%)	15 (15.46 within PRIME-NL region)
Reported no-change in PRIME-NL region—no. (%)	24 (24.74 within PRIME-NL region)
Reported increase in PRIME-NL region—no. (%)	58 (59.79 within PRIME-NL region)
Reported decrease in usual care region—no. (%)	85 (32.32 within usual care region)
Reported no-change in usual care region—no. (%)	82 (31.18 within usual care region)
Reported increase in usual care region—no. (%)	96 (36.50 within usual care region)

Note. *The healthcare innovation project called PRIME Parkinson Evaluation Study program is nationwide prospective evaluation in the Netherlands. The program assesses the impact of the innovative PRIME Parkinson care model, which was progressively implemented in Central Gelderland beginning in 2021. This care model's outcomes are compared with those from other regions where usual care models are in place. The table presents regional differences by showing absolute numbers and percentages of reported decreases, no changes, and increases.

Table S8. Item structure for calculating Hoehn & Yahr (HY) score.

HY-score	Description	Questionnaire	Item	Criteria
**HY 5**	wheelchair bound or bedridden unless aided	P9: UPDRS part II	walking and balance	*"I usually need support from someone to walk safely without falling."*
**HY 4**	severe disability; still able to walk or stand unassisted	P9: UPDRS part II	getting out of bed, out of a car, or standing up from a deep chair	*"I usually or always need help."*
		P9: UPDRS part II	getting out of bed, out of a car, or standing up from a deep chair	*"Getting out of bed, out of a car, or standing up from a deep chair."*
		P9: UPDRS part II	walking and balance	*"I usually use an aid (cane, walker) to walk safely without falling. However, I usually do not need someone to support me."*
**HY 3**	mild to moderate bilateral disease; some postural instability; physically independent	P9: UPDRS part II	getting out of bed, out of a car, or standing up from a deep chair	*"I need more than one attempt to get up, or I sometimes need help with it."*
		P9: UPDRS part II	walking and balance	*"I sometimes use an aid to walk, but I do not need any help from someone else."*
**HY 2**	bilateral involvement without impairment of balance	P9: questionnaire motor symptoms	bradykinesia left/right	left <10 & right <10
**HY 1**	unilateral involvement only	P9: questionnaire motor symptoms	bradykinesia left/-right	*"Having completed the three relevant questionnaires but not fulfilling any of the abovementioned criteria."*

Note. The HY-score was based on six questions from the Movement Disorder Society Unified Parkinson's Disease Rating Scale (MDS-UPDRS) and two self-structured items.

Supplementary Figures

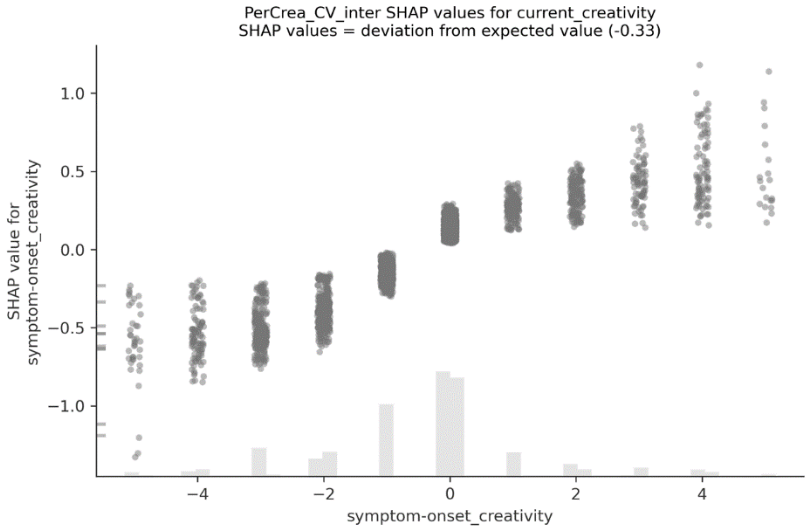

Figure S1. Contribution of 'symptom-onset_creativity' in predicting the target.

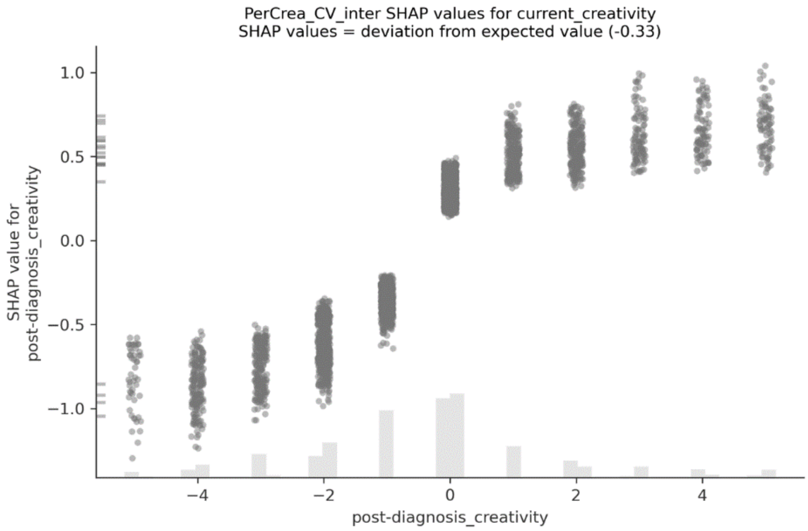

Figure S2. Contribution of 'post-diagnosis_creativity' in predicting the target.

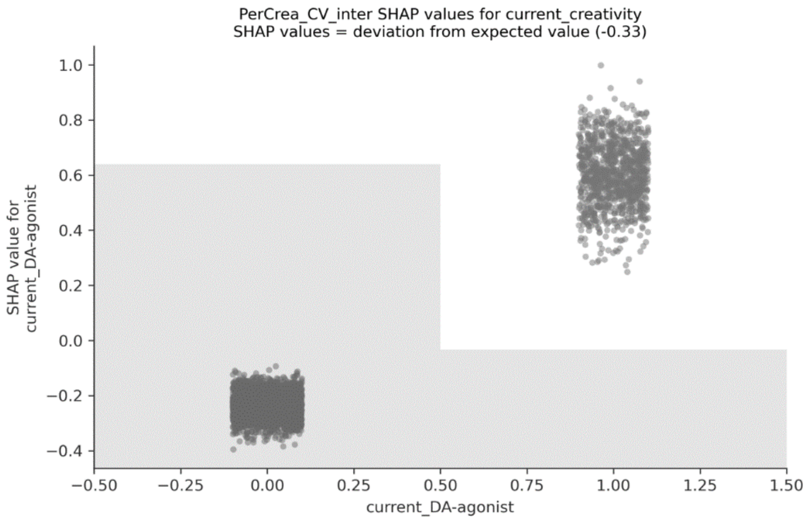

Figure S3. Contribution of 'current_DA-agonists' in predicting the target.

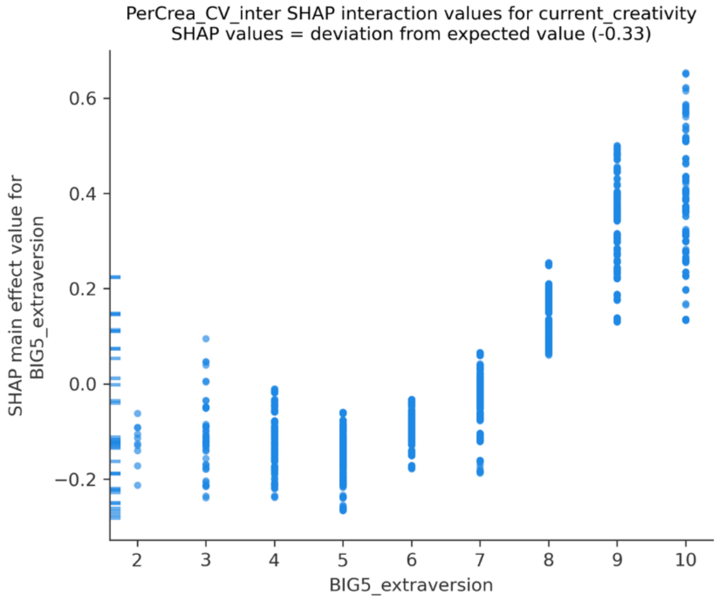

Figure S4. Contribution of 'BIG5_extraversion' in predicting the target.

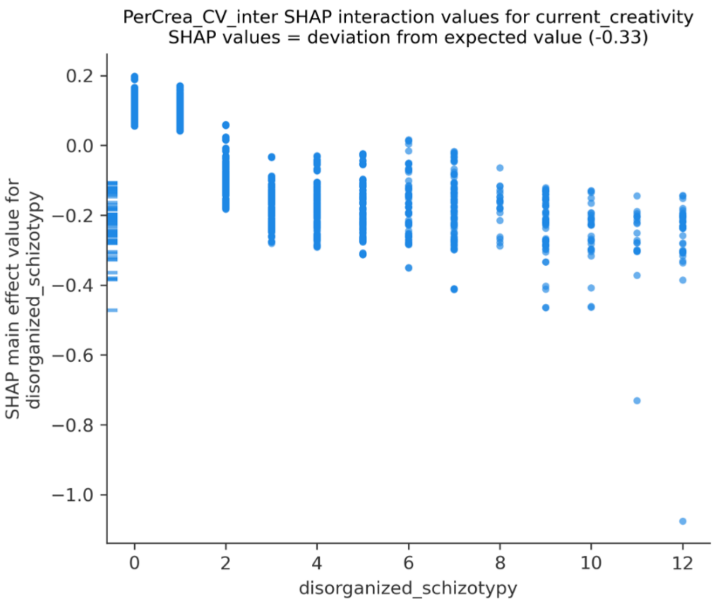

Figure S5. Contribution of 'disorganized_schizotypy' in predicting the target.

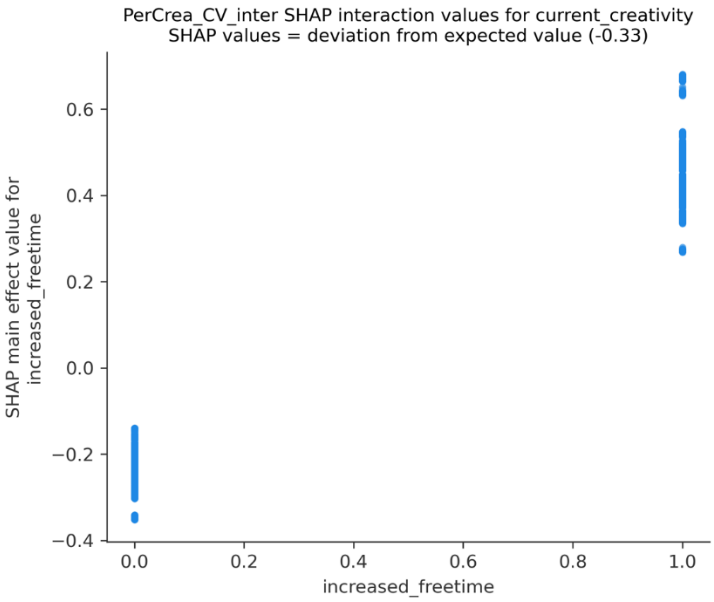

Figure S6. Contribution of 'increased_freetime' in predicting the target.

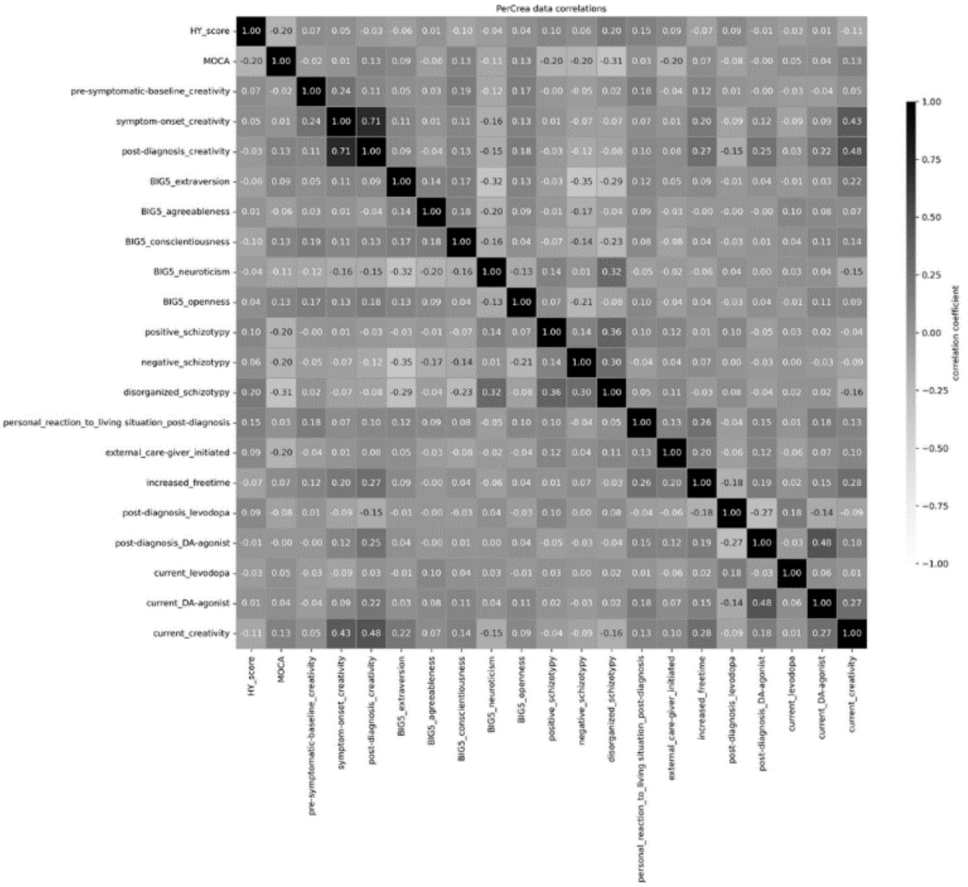

Figure S7. Correlation heatmap
